# Contemplating IL‐6, a double‐edged sword cytokine: Which side to use for stroke pathology?

**DOI:** 10.1111/cns.14041

**Published:** 2022-12-07

**Authors:** Molly Monsour, Davide M. Croci, Siviero Agazzi, Cesar V. Borlongan

**Affiliations:** ^1^ University of South Florida Morsani College of Medicine Tampa Florida USA; ^2^ Department of Neurosurgery and Brain Repair University of South Florida, Morsani College of Medicine Tampa Florida USA; ^3^ Center of Excellence for Aging and Brain Repair University of South Florida Morsani College of Medicine Tampa Florida USA

**Keywords:** hemorrhagic stroke, interleukin 6, ischemic stroke, signaling

## Abstract

Interleukin (IL)‐6 is a unique cytokine due to its dual signaling, with one pathway being pro‐inflammatory (trans) and the other homeostatic (classical). Both of these pathways have been implicated in neuroinflammation following stroke, with initial inflammatory mechanisms being protective and later anti‐inflammatory signaling promoting ischemic tissue recovery. IL‐6 plays a major role in stroke pathology. However, given these distinctive IL‐6 signaling consequences, IL‐6 is a difficult cytokine to target for stroke therapies. Recent research suggests that the ratio between the pro‐inflammatory binary IL6:sIL6R complex and the inactive ternary IL6:sIL6R:sgp130 complex may be a novel way to measure IL‐6 signaling at different time points following ischemic injury. This ratio may approximate functional consequences on individualized stroke therapies, allowing clinicians to determine whether IL‐6 agonists or antagonists should be used at specific time points.

## Graphical abstract

*IL*‐*6 mounts a balancing act on stroke inflammation.* IL‐6 employs discrete pathways that elicit divergent inflammatory responses. Interrogating the role of exogenous sgp130 may lead to optimal modulation of the inflammatory signaling pathway, allowing IL‐6 to exert therapeutic effects in stroke.

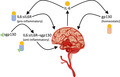

## 1 INTRODUCTION

Ischemic stroke (IS) and hemorrhagic (HS) stroke are incredibly detrimental and in need of novel treatment development. HS, accounting for 15% of strokes,^1^ involves blood vessel damage, such as berry aneurysms, and their subsequent rupture.^2^ 50% of patients with HS die or suffer from compromised cognitive abilities, functionality, and quality of life scores.^3^ Unfortunately, there are no treatments for HS.^4^ Ischemic stroke (IS), caused by a lack of blood flow to the brain due to vascular blockage, has only slightly more promising outcomes. 85% of strokes are caused by ischemia, and 25% of patients die from IS.^1^, ^5^ While thrombolytic therapies, such as tissue plasminogen factor (tPA) and mechanical thrombectomy (MT) can be used for IS, they are accompanied by a high risk for hemorrhagic transformation.^6^ Furthermore, these therapies must be employed in limited time windows or can be more harmful than helpful.^7^, ^8^, ^9^, ^10^, ^11^ IS and HS have various methods of cell death, including excitotoxicity, oxidative stress, free radical accumulation, mitochondrial dysfunction, impaired neurogenesis, angiogenesis, vasculogenesis, and inflammation.^12^, ^13^, ^14^ Regarding inflammatory processes following stroke, IL‐6 plays a significant role in IS and HS pathophysiological processes. In the early stages following stroke, IL‐6 is vital in inducing an inflammatory cascade and eventually inducing the activity anti‐inflammatory mediators.^15^, ^16^ Later, leukemia inhibitory factor and ciliary neurotrophic factor, members of the IL‐6 family, are necessary to stimulate neurogenesis, cell survival, and stem cell proliferation.^15^, ^17^ These IL‐6 cytokine family members work via different signaling mechanisms, and maintaining a balance of these pathways plays a considerable role in neuroinflammation.

IL‐6 possesses two unique signaling pathways with widely variable outcomes (Graphical Abstract). Classical signaling uses membrane‐bound IL‐6 receptors. These IL‐6 receptors are not present in neuroinflammatory cells; thus, classical signaling propagates a homeostatic, anti‐inflammatory signaling pathway.^18^, ^19^ Once IL‐6 is bound, gp130, the signal‐transducing protein, is dimerized and signals are sent via the JAK/STAT3 pathway to promote homeostasis in response to immune system stimulation.^20^, ^21^, ^22^ Classical signaling is seen in nerve regeneration after trauma and spinal cord injuries.^23^, ^24^, ^25^ Contrarily, the trans pathway is pro‐inflammatory due to the activation of gp130 on neuroinflammatory cells.^18^ Gp130 is present in neurons, neuronal cells, endothelial cells, and oligodendrocytes.^26^ Trans‐signaling uses a soluble IL‐6 receptor (sIL6R), forming the binary IL6:sIL6R complex, and later activating gp130.^18^ This binary complex is pro‐inflammatory; however, soluble gp130 (sgp130) can block inflammation by establishing a ternary IL6:sIL6R:sgp130 complex. This ternary complex prohibits gp130 binding and IL‐6 trans‐signaling. Obtaining a greater understanding of each of these signaling mechanisms and their roles in neuronal pathologies, such as stroke, could revolutionize treatment options. Recently, the utility of the binary/ternary (B/T) ratio in measuring IL‐6 inflammatory signaling to predict ischemic stroke has been elucidated.^27^ The B/T ratio may be a novel option to optimize IL‐6 targeted therapies for stroke, and further investigation should be conducted to better understand its clinical relevance in stroke.

## 2 A NOVEL BIOMARKER DISCOVERY

To examine the role of IL‐6 trans‐signaling in propagating IS secondary to atrial fibrillation (AF), the ratio between pro‐inflammatory binary IL6:sIL6R complex to the inactive ternary IL6:sIL6R:sgp130 complex (B/T ratio) in 4232 60‐year‐old patients was examined.^27^ Over a 20‐year period, 203 of these participants suffered from IS. Twenty‐nine patients had pre‐existing AF, while 279 patients were diagnosed with AF over the course of the study. AF was positively correlated to IS incidence, but B/T ratio was not different among those who had AF and IS versus those who had AF but no IS. B/T ratio was, however, significantly related to IS risk regardless of AF, as those who had a stroke during follow‐up had higher baseline B/T ratios. Contrarily, AF was not related to higher B/T ratios, but generally higher IL‐6 levels did correlate with greater AF incidence and shorter time to AF diagnoses. Ultimately, these researchers suggest that the B/T ratio may be a novel biomarker to determine patients at risk for IS secondary to vascular pathology, but not secondary to AF‐induced emboli. Our group aims to further analyze the use of the B/T ratio as a biomarker, while also employing this ratio to optimize and personalize IL‐6 trans‐signaling targeted therapies for IS and HS.

## 3 A FAVORABLE END OF THE DOUBLE‐EDGED SWORD?

IL‐6 expression levels are increased following IS and HS^28^, ^29^; however, there are conflicting reports of whether blocking IL‐6 signaling is beneficial or detrimental following stroke. Blocking IL‐6 signaling affords beneficial effects in many stroke studies. In HS, peroxisome proliferator‐activated receptor‐γ agonists can reduce IL‐6 mRNA in mouse models of intracranial aneurysms, ultimately reducing rupture occurrence.^30^ Inhibiting activator protein 1 (AP‐1), a gene regulator known to increase cytokine production, with SR11302 in mice models of HS also shows reduced IL‐6 levels, which may be related to the reduced brain injury and toxic inflammation following HS after treatment.^31^ Treatment with Tocilizumab, an IL‐6 receptor antagonist, reduces the occurrence of vasospasm, an injurious consequence of HS,^32^ and mitigates cell death in rabbit models of HS.^33^ Similarly, Tocilizumab reduces brain atrophy, mortality, and functional deficits in mouse models of IS.^34^ Using cell‐based therapies, IS rat models treated with Roxadustat‐prepped bone marrow stromal cells have decreased infarct volumes and improved behavior recovery paralleling reduced pro‐inflammatory cytokines, including IL‐6.^35^ Similarly, human embryonic stem cell‐derived and umbilical cord MSCs decrease IL‐6 mRNA in murine models of middle cerebral artery occlusion. This decrease in IL‐6 may be relevant to the increased neurogenesis, increased angiogenesis, and decreased apoptosis seen in these treated animals.^36^, ^37^ Given the role of IL‐6 in inflammation following stroke, it is understandable that blocking this cytokine prior to potential exacerbated inflammation offers therapeutic benefit. Blocking IL‐6 to exert therapeutic effects may be highly time‐dependent (i.e., acute phase of stroke), complicating the treatment regimen.

Various studies have contradicted the aforementioned therapeutic approaches of blocking IL‐6 signaling, suggesting that IL‐6 amplification is also helpful in mitigating stroke damage. In IL‐6 knockout mice models of IS, exogenous administration of IL‐6 induces poststroke angiogenesis. Without IL‐6, angiogenic mediators are diminished and mice exhibit larger infarct volumes with poor revascularization. Treatment with IL‐6, however, increases the transcription of angiogenic genes.^38^ In rodent models, IL‐6 injection after ischemic injury reduces cell death, learning disabilities, and infarct damage.^39^, ^40^ Blocking IL‐6 receptors with a monoclonal antibody in rat models of middle cerebral artery occlusion leads to high levels of apoptosis and large infarct volumes.^41^ IL‐6 is also important for hematopoietic stem cell differentiation, and stem cells have been proposed as a revolutionary treatment for stroke.^12^, ^42^, ^43^ Indeed, bone marrow‐derived stem cells (BMSCs) amplify regulatory T‐cell (Treg) proliferation in ischemic tissue after IS, promoting anti‐inflammation and neuroprotection in neuronal cell cultures. The mechanism of action for these protective qualities of BMSCs may involve IL‐6, as increased Treg concentration correlates with increased IL‐6 secretion by BMSCs.^44^ Preconditioning of mesenchymal stem cells with IL‐1β and IFN‐γ enhances IL‐6 production, ultimately increasing anti‐inflammatory macrophage differentiation.^45^ The therapeutic benefits of elevated IL‐6 after IS can also be seen using epidermal neural crest stem cells.^46^ Peripheral inflammation also contributes significantly to stroke pathology.^47^ Administration of partial MHC class II construct, DRmQ, to rat models of IS, reduces splenic contributions to neuroinflammation, including amplifying splenic IL‐6 production, and decreases stroke pathogenesis. Future research should also aim to compare the therapeutic effectivity of direct IL‐6 application versus BMSC‐induced IL‐6 production for eventual clinical translation. Other cytokines, such as IL‐13 and IL‐4, may play a vital role in IL‐6 signaling as well. IL‐13 and IL‐4 have been shown to increase IL‐6 production,^48^ and elevations in both IL‐13 and IL‐4 demonstrate reparative effects following stroke in mice models.^49^, ^50^ There is less research regarding the amplification of IL‐6 following HS; however, given similar inflammatory responses between HS and IS, further investigation is needed.^51^ In addition to blocking IL‐6 signaling, amplification of IL‐6 may offer equally therapeutic benefits to improving stroke prognoses, depending on the time course and each patient's unique inflammatory responses. The fine balance between amplifying or inhibiting specific IL‐6 pathways creates a complicated situation for treatment design. Due to IL‐6's dominant role in stroke pathology, however, it is vital to understand and specifically target the dynamic IL‐6 signaling pathways that may confer deleterious or beneficial stroke outcomes.

## 4 CLINICAL MILESTONES

While the preclinical studies are numerous, clinical studies are less prevalent. Genetic contributions to IL‐6 levels are significant in predicting stroke occurrence and prognoses, with lower genetically induced production of IL‐6 being protective against IS. Interestingly, those predisposed to lower IL‐6 production also showed greater sIL6R levels.^52^ Some research shows higher IL‐6 serum levels at study enrollment correlate to worse outcomes at 3 and 6 months (NCT01953549).^53^ Elevations in IL‐6 at least 3 weeks after initial lacunar stroke predict recurrent vascular events, such as stroke, vascular death, and myocardial infarction.^54^ Lacunar strokes are commonly due to hypertension, however, which is a major confounding factor of these secondary events. Regarding HS, higher IL‐6 levels at admission are related to worse prognosis regarding function, ICH volume, and edema.^55^ Importantly, all these studies measure initial IL‐6 concentrations long after stroke incidence; however, the pro‐inflammatory or anti‐inflammatory impacts of IL‐6 vary significantly depending on the stroke recovery timeline.^15^, ^16^ While these studies suggest IL‐6 is a detrimental cytokine for stroke prognosis, it rather strengthens the need to better understand the temporality in signaling mechanisms of IL‐6 following stroke.

Regarding attempts to resolve functional deficits following stroke, some report measures of IL‐6. Statin administration, a common treatment for dyslipidemia to reduce stroke risk, is related to improved prognosis and lower inflammatory markers, including IL‐6 (NCT02225834).^56^ Another study employed an IL‐1 receptor antagonist in patients 5 h after IS. IL‐1 induces IL‐6 expression, thus, blocking this signaling reduces plasma IL‐6 as well. This antagonism did not, however, show any clinical benefit (ISRCTN74236229).^57^ Similarly, oleoylethanolamide shows reduced inflammation, oxidative stress, and dyslipidemia after ischemic stroke, but no functional recovery is reported.^58^

Ongoing clinical trials are measuring IL‐6 in relation to ischemic stroke prognosis (NCT05004389; NCT03297827), ischemic stroke treatment efficacy (NCT04705779), hemorrhagic stroke recovery using in‐bed cycle ergometry (NCT04027049), and even IS and HS recovery following autologous mesenchymal stem cell therapy (NCT04063215).

## 5 FUTURE DIRECTIONS

Given the acute role of IL‐6 in inflammation, and the delayed neurotrophic role of IL‐6,^15^ we propose to probe the ideal time to enhance or inhibit the IL‐6 trans, inflammatory signaling pathway. It is likely that optimal IL‐6 targeted therapies modulate the trans pathway to induce trans‐signaling early on but mitigate this pathway later to avoid amplified neuroinflammatory responses.^59^ To achieve this balance, sgp130 antibodies can be used to aid in ternary complex formation, ultimately enhancing the IL6:sIL6R:sgp130 trans‐signaling antagonism in clinical and preclinical studies of vascular disease.^60^ The question of ideal timing for therapeutic effect, however, remains to be elucidated. Considering recent literature,^27^ we suggest the use of the B/T ratio to determine when the body is naturally amplifying versus lessening inflammatory IL‐6 signaling. With this knowledge, IL‐6 agonists can be used while the B/T ratio is high to support the body's natural mechanisms. Once the B/T ratio lowers, however, a switch to IL‐6 trans‐signaling antagonist therapies, such as sgp130, can be given to support the body's attempts to lessen inflammation. The use of each patient's B/T ratio is critical, based on the natural variation in people's bodily responses. By adjusting pharmaceutic intervention targeting IL‐6 trans‐signaling, clinicians may be able to aid in the body's natural flipping of the double‐edged sword. Further research is needed, however, to enhance understanding of the B/T ratio following stroke and examine how IL‐6 signaling interventions should be administered in relation to this ratio.

## 6 CONCLUSION

Drawing on recent findings,^27^ we encourage researchers to study the use of the B/T ratio following IS or HS to determine whether IL‐6 trans‐signaling should be amplified or mitigated. This novel approach to pharmaceutical intervention of inflammation allows for tailored treatment for stroke patients. Recognizing the key role of IL‐6 in stroke pathogenesis, alongside its complicated signaling mechanisms, proceeding with individualized analysis of time‐dependent predominant signaling pathways in each patient prior to initiating IL‐6‐based treatments may aid to achieve the cytokine's therapeutic outcomes in stroke.

## AUTHOR CONTRIBUTIONS

M.M. and C.V.B. wrote the main manuscript. M.M. and C.V.B. designed the figure. S.A. and D.C. reviewed the manuscript and figure.

## FUNDING INFORMATION

C.V.B. was funded by the National Institutes of Health (NIH) R01NS090962, NIH R01NS102395, and NIH R21NS109575. Additionally, C.V.B. was funded and received royalties and stock options from Astellas, Asterias, Sanbio, Athersys, KMPHC, and International Stem Cell Corporation and has also received consultant compensation from Chiesi Farmaceutici. C.V.B. also declares patents and patent applications related to stem cell therapy.

## CONFLICT OF INTEREST

C.V.B. is an Editorial Board member of CNS Neuroscience and Therapeutics and a co‐author of this article. To minimize bias, they were excluded from all editorial decision‐making related to the acceptance of this article for publication.

## Data Availability

No data availability statement is applicable.
